# Novel Poly(3-hydroxybutyrate-g-vinyl alcohol) Polyurethane Scaffold for Tissue Engineering

**DOI:** 10.1038/srep31140

**Published:** 2016-08-09

**Authors:** Adriana Pétriz Reyes, Ataúlfo Martínez Torres, Ma. del Pilar Carreón Castro, José Rogelio Rodríguez Talavera, Susana Vargas Muñoz, Víctor Manuel Velázquez Aguilar, Maykel González Torres

**Affiliations:** 1 Laboratorio de Neurobiología Molecular y Celular, Instituto de Neurobiología, Universidad Nacional Autónoma de México, Querétaro 76230, México; 2 Instituto de Ciencias Nucleares, Universidad Nacional Autónoma de México, México D.F, 04510, México; 3 Centro de Física Aplicada y Tecnología Avanzada, Universidad Nacional Autónoma de México, Querétaro 76230, México; 4 Facultad de Ciencias, Universidad Nacional Autónoma de México, México D.F, 04510, México

## Abstract

The design of new synthetic grafted poly(3-hydroxybutyrate) as composite 3D-scaffolds is a convenient alternative for tissue engineering applications. The chemically modified poly(3-hydroxybutyrate) is receiving increasing attention for use as biomimetic copolymers for cell growth. As of yet, these copolymers cannot be used efficiently because of the lack of good mechanical properties. Here, we address this challenge, preparing a composite-scaffold of grafted poly(3-hydroxybutyrate) polyurethane for the first time. However, it is unclear if the composite structure and morphology can also offer a biological application. We obtained the polyurethane by mixing a polyester hydroxylated resin with polyisocyanate and the modified polyhydroxyalkanoates. The results show that the poly(3-hydroxybutyrate) grafted with poly(vinyl alcohol) can be successfully used as a chain extender to form a chemically-crosslinked thermosetting polymer. Furthermore, we show a proposal for the mechanism of the polyurethane synthesis, the analysis of its morphology and the ability of the scaffolds for growing mammalian cells. We demonstrated that astrocytes isolated from mouse cerebellum, and HEK293 can be cultured in the prepared material, and express efficiently fluorescent proteins by adenoviral transduction. We also tested the metabolism of Ca^2+^ to obtain evidence of the biological activity.

The amazing structural diversity of polyesters has inspired researchers to continue the discovery of new alternatives for tissue engineering applications^1^,^2^,^3^,^4^,^5^,^6^. Polyhydroxyalkanoates have stood out in this category driven by their good properties for use in biomedicine such as biocompatibility and biodegradability^7^. They have attracted widespread interest for diverse applications, including the fracture repair, implants, controlled released systems and the preparation of scaffolds^8^,^9^,^10^. Poly(3-hydroxybutyrate), which is called the first member of this “green” polymers’ family can be synthesised biologically by a large number of bacteria^11^. It is undoubtedly a promising biomaterial widely used in medical science because of its non-toxicity controllable degradation and thermoplasticity^12^.

Previous studies indicated that P(3HB), and its copolymers were also used to prepare composite scaffolds^13^,^14^,^15^,^16^. In general terms, the preparation of scaffolds from a biomaterial has gained increasing attention because of its versatility and importance^17^,^18^,^19^,^20^,^21^. Regarding P(3HB) scaffolds, several methods involving their preparation were reported, such as electrospinning, salt-leaching, blend nanofibers, hollow fibers, nanofibrous electrospun, porous composite, ceramic reinforcement and hybrid scaffold systems^22^,^23^,^24^,^25^,^26^,^27^,^28^,^29^,^30^,^31^,^32^,^33^. Despite its many attributes, the P(3HB) intrinsic properties are limited. The main drawbacks are the hydrophobicity, surface chemical inactivity and the lack of functional groups^34^. Consequently, many efforts have been conducted to modify the P(3HB) structure to amplify its application^35^,^36^,^37^. The use of chemicals initiating agents for P(3HB) transformation implies dealing with residuals. Hence, the use of gamma irradiation rather than chemicals to modify its structure is more suitable because it allows obtaining pure graft copolymers with simplicity of operation^28^,^38^.

Recently, we prepared an interesting copolymer by gamma-radiation-induced grafting of vinyl acetate onto P(3HB). The product obtained was hydrolysed to yield poly(3-hydroxybutyrate) grafted with poly(vinyl alcohol) (P(3HB-g-VA))^39^. Surprisingly, this copolymer showed the ability to produce electricity by reorientation of the molecules with gradual stress compression^40^. It is known that this grafted P(3HB) is also biodegradable and biocompatible, which could be used to prepare nanoparticles with potential application as drug delivery systems^41^. Therefore, we proposed the use of P(3HB-g-VA) for the synthesis of a polyurethane foam scaffold. The lack of existing research on the fabrication of these materials prompted us to study their synthesis in depth. It is not yet known if this type of polyurethane can be successfully obtained and used for biomedical purposes.

Here, we describe for the first time a novel method in which a grafted P(3HB) is combined with a polyester hydroxylated resin and poly-isocyanate to yield chemically-crosslinked polyurethane. Our strategy relies on adding the P(3HB) grafted with poly(vinyl alcohol) as a chain extender in a presence of a porogen to prepare a foam scaffold. This approach enabled the evaluation of the activity of mammalian cells on the polymeric structure. To the best of our knowledge, this research constitutes the first of its kind, in which a gamma radiation-induced P(3HB) graft copolymer is successfully used to synthesise a polyurethane scaffold. We also report a proposal for the polymerisation mechanism and demonstrate the great potential of this structural component in tissue engineering.

## Results

### Synthesis and characterisation of the P(3HB-g-VA) polyurethane scaffold

**Figure 1:**
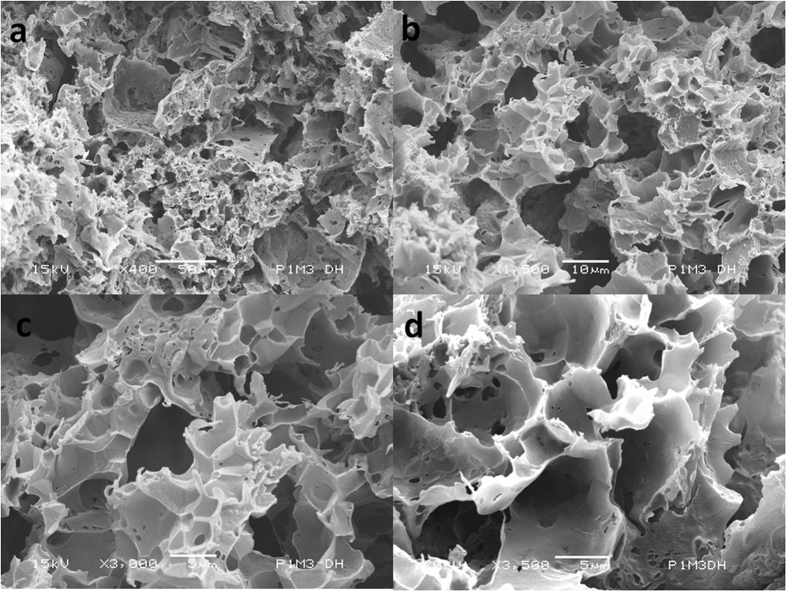
Scanning electron microscope images of the cross-section of the P(3HB-g-VA) polyurethane scaffold with (**a**) ×400; (**b**) ×1000; (**c**) ×3000; and (**d**) ×3500 magnification.

We prepared round shape scaffolds of roughly 10 mm in diameter and 2.5 mm in height, with an average dry weight of 525 ± 3 mg. The scaffolds, hereafter called P1M3DH, displayed a mean compressive modulus and compressive strength of 20 ± 2 and 2 ± 0.1 MPa respectively (p < 0.05). Figure 1a–d present the scanning electron microscope (SEM) micrographs of the cross-section of the P(3HB-g-VA) polyurethane scaffold at different magnifications. The cross-section SEM images revealed a porous structure with pore size ranging from 1 to 10 μm and average porosity of approximately 92 ± 2%. The magnified view of the surface showed a rough morphology divided into three main areas. The first region consisted of an open non-directional network of pores, with average pore size ranging from 5 to 10 μm. The multi-scale pore structures are caused by the salt leaching. The second area displayed uneven porosities of less than 5 μm pore size. This area showed a high degree of inter-connectivity along with the third area. The latest area exhibited a rounded geometry and suggested a random non-directional pore structure, which ranges in size, of approximately 1 ± 0.14 μm. Therefore, owing the large degree of interconnectivity and the wide range of macro-porosity, it appears that the P(3HB-g-VA) polyurethane scaffold may be suitable for the cell growth and proliferation^12^,^16^,^22^,^25^,^28^,^31^,^42^,^43^,^44^,^45^,^46^,^47^,^48^,^49^. On the other hand, the result of the mechanical properties of P(3HB-g-VA) polyurethane scaffolds is consistent with that obtained for the synthesis of poly(3-hydroxybutyrate) and nanohydroxyapatite thermal remolded composite scaffold and three-dimensional scaffolds prepared from lyophilized poly(3-hydroxybutyrate-co-hydroxyhexanoate)^44^,^49^.

One can expect that the compressive strength should have a greater result, but it is strongly influenced by the high porosity formed in the leaching process and the brittleness of P(3HB-g-VA)^40^. In addition, porosity testing for P1M3DH scaffolds revealed that the porosity of the samples increased as the concentration of the copolymer increased. Consequently, the compressive strength decreased with increasing weight percentage of P(3HB-g-VA) in the prepared dough.

### A proposed mechanism for the preparation of P(3HB-g-VA) polyurethane scaffold

The mechanism by which the three-component polyurethane forms a 3D-scaffold is proposed. First, the resin (aliphatic isocyanate) is mixed with the hardener (polyol). In this step, the 1,4-Diisocyanatobutane (BDI) is attacked by the polyethylene oxide (PEO) molecules yielding ionic species (i). Then, the PEO hydrogen is swiped by the BDI nitrogen (ii). The ii species can react with BDI by another alcohol group to end up with two isocyanate groups (iii). This product is called the prepolymer. The prepolymer is in this case a urethane dimer intermediate. In the next step (chain extension), it can react with a chain extender to yield the final polyurethane. It seems that there are two main chain extenders, the polyol and the hydroxylated polyester (P(3HB-g-VA). A linear segmented material is produced by the first one (iv). A chemically crosslinked polyurethane is obtained by the polyester (v). This reaction is known as the prepolymer process.

**Figure 2:**
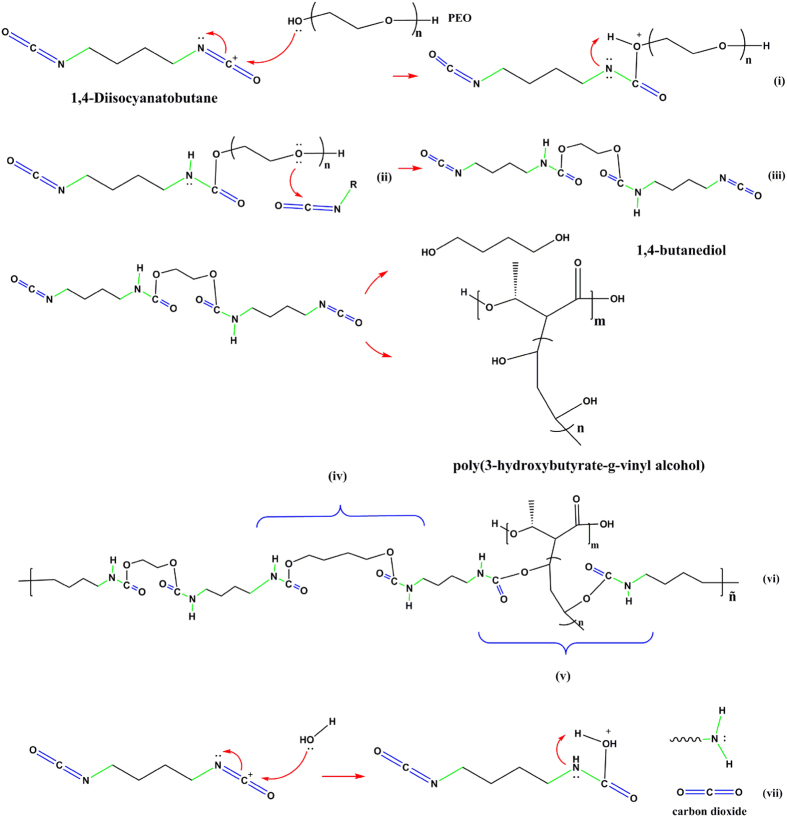
Proposed mechanism for the P(3HB-g-VA) polyurethane synthesis.

Additionally, the formation of the P(3HB-g-VA) polyurethane foam (see Fig. 2) should be attributed to two main mechanisms. The first one is the addition of a porogen, in this case sodium acetate (NaAc). This salt is randomly aggregated three-dimensionally until a high pore volume is reached. The second mechanism involves the reaction of water molecules to the isocyanate to yield urea and carbon dioxide (vii).

As a result a complex chemically-crosslinked structure is achieved (vi). The novel structure contains a soft segment formed by the prepolymer, and two arbitrary segments from the chain extenders (1,4-Butanediol (BDO) and P(3HB-g-VA)), which are called hard segments. The foam structure depends on the BDI/ PEO/BDO/P(3HB-g-VA) equivalent ratio as well as the porogen size and the quantity that was added to the mixture. The mixture is cured in a mould, where the polyurethane hardens to form a thermoset. The NaAc is leached by Soxhlet extraction in water after the product is cured. Consequently, three-dimensional pores of up to 10 μm are formed.

### Astrocytes and HEK293 cells grown in P1M3DH scaffolds

**Figure 3:**
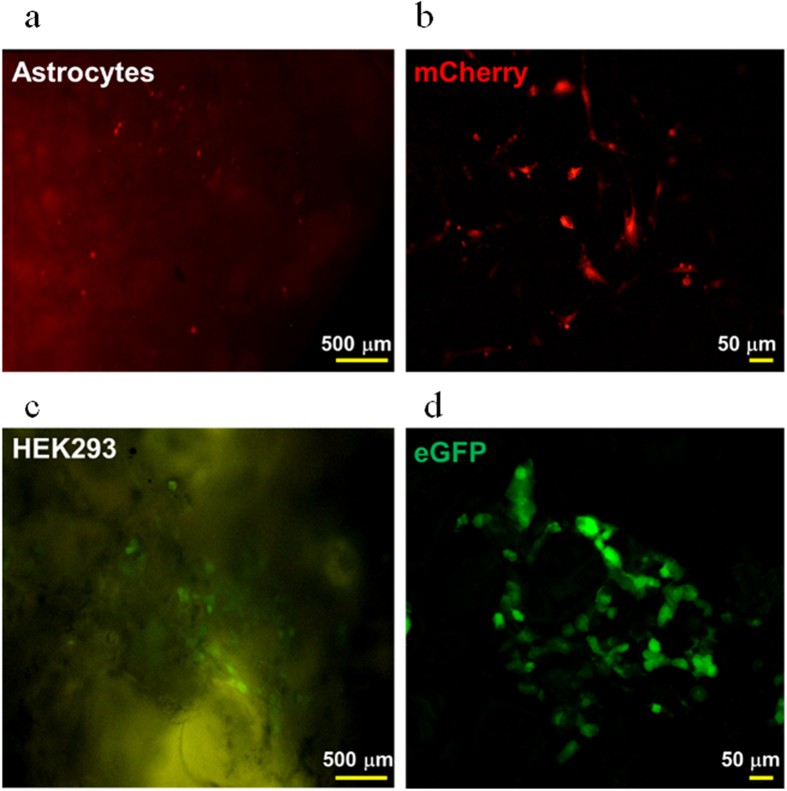
Astrocytes and HEK293 cells grown on P1M3DH. **(a)** Epifluorescence microscopy of cerebellar astrocytes expressing mCherry. (**b)** Laser confocal image of astrocytes grown on P1M3DH. (**c)** Epifluorescence microscopy of HEK293 cells expressing eGFP grown on P1M3DH scaffolds. (**d)** Laser confocal image of HEK293 cells on P1M3DH. Notice that the autofluorescence emitted by the scaffold under the epifluorescence microscope is eliminated by proper filtering under the confocal microscope. Astrocytes imaged in B show complex morphologies, from two to multiple processes. Dark areas on B and D correspond to deep cavities on the surface of the scaffold, as those revealed by SEM.

To test the ability of P1M3DH scaffolds for growing mammalian cells, we cultured astrocytes isolated from mouse cerebellum and HEK293 (Fig. 3a,b). After three days *in vitro* we imaged the cells expressing either the fluorescent protein mCherry (Fig. 3a,b) or eGFP (Fig. 3c,d). From the first day, the cells attached well to the surface of P1M3DH and adapted to the rough surface of the scaffold; by the second day both cell types developed complex processes normally observed *in vivo*, and in many cases the cells touched each other forming clumps associated with the cavities of the scaffold surface. When the fluorescence emitted by the cells was observed in an epifluorescence microscope it was evident that much of the surface of the scaffold generated autofluorescence (Fig. 3a,c); this autofluorescence was efficiently filtered when observed by confocal microscopy (Fig. 3b,d), which revealed the complexity of the cell morphologies.

### Calcium imaging in HEK293 cells grown in P1M3DH scaffolds

In the previous experiments it was shown that mammalian cells grow efficiently on P1M3DH and express efficiently fluorescent proteins by means of adenoviral transduction. This appeared to prove that astrocytes and HEK293 cells are physiologically active when grown on P1M3DH. However, to have clear-cut evidence of their biological activity we tested the metabolism of Ca^2+^, a cellular second messenger necessary for vital metabolic cascades.

**Figure 4:**
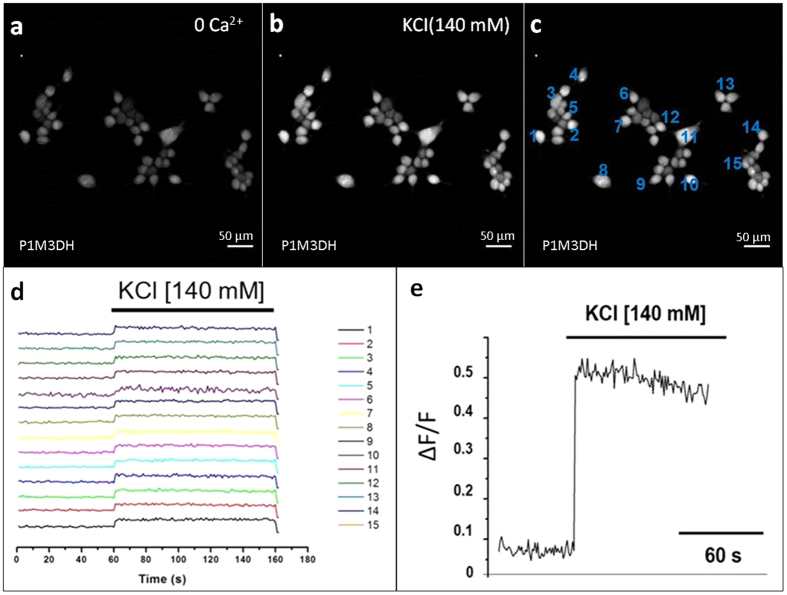
KCl increases Fluo-4AM fluorescence intensity in HEK293. **(a)** Image of HEKs loaded with Fluo-4AM grown on the P1M3DH calcium free solution. (**b**) Increased fluorescence intensity after hyperpolarising the cells with high potassium solution (140 mM KCl). (**c**) Randomly selected cells for labeling of ROIs. (**d)** Ratio of fluorescence intensity change (∆F/F) in cells labeled in C. (**e)** Mean of fluorescence change (∆F/F).

Figure 4a shows that HEK293 cells incorporated the fluorescent Ca^2+^ indicator Fluo-4AM. This was considered as the basal activity of Ca^2+^ in these cells and was recorded for 60 sec. If, as suggested by previous observations, the cells were metabolically active, a sudden rise of fluorescence intensity should be detected when challenged by changes in the plasma membrane resting potential. This was proven when high potassium was added to the cellular medium (Fig. 4b); in this image the increased fluorescence showed up quite clearly in most of the cells attached to P1M3DH. Fifteen of these cells were randomly chosen for quantitative analysis (Fig. 4c) and ∆F/F plotted as a reason of time. ∆F/F recordings from individual cell are shown in Fig. 4d and plotted in Fig 4e. On average, the cells emitted five times more fluorescence when exposed to high potassium, thus unequivocally indicating that HEK293 cells are physiologically active. Supplementary video illustrates the time-curse of this protocol (see Video 1).

## Discussion

The structure of P(3HB) molecules is very inactive, with lack of functional groups, bringing a hydrophobic nature. The introduction of polyvinyl alcohol groups to the polyester structure provides a greater hydrophilicity, biodegradability, and also functionality, as demonstrated in our previous works^39^,^40^,^41^. This strategy promotes a new alternative for application. In this case, the PVA moieties can be used to generate a crosslinked reaction that creates a modern kind of polyurethane. This approach paves the way for the synthesis of a novel family of thermosets with potential applications in tissue engineering. The combination of different polyisocianates, prepolymers, chain extenders, porogens and gamma-radiation-induced grafted polyhydroxyalkanoates bring about a large new family of what we herein define as “polyurethanoates”. We demonstrated that our first attempt succeeded in improving the mechanical properties of the scaffolds and the obtaining of an appropriate morphology, which allows the attachment of the mammalian cells as a prerequisite for evaluating the biocompatibility. Additionally, it was unequivocally demonstrated that the attached cells were physiologically active. By careful manipulation, the pore architecture of the scaffolds can be controlled and therefore, the surface presentation can be improved. The interconnected porous structure suggests a favourable environment for ingrowth of cells, vascularisation and the diffusion of nutrients for cell proliferation. The experiments have focused on the research of the P(3HB-g-VA) polyurethane scaffold *in vitro*, but further research should be performed on evaluating the novel scaffolds *in vivo*. Nevertheless, the advancement in tissue engineering technology for the study of Astrocytes and HEK293 cells grown involves increasing the knowledge about the neural connections and gene expression respectively. This research reveals a promising candidate for tissue engineering and shows the way for the discovery of novel chemical structures that enables to continue growing the diversity of design in advanced biomaterials systems^50^,^51^.

## Methods

### Fabrication and characterisation of the P(3HB-g-VA) polyurethane scaffold

The synthesis of P((3HB)-g-VA) was carried out through the simultaneous irradiation method. P(3HB) and vinyl acetate (VAc), were subjected to the same source of ^60^Co-gamma-radiation in air (Gamma Beam 651 PT, Nordion International), which has a dose rate of about 1 kGy/h and a dose of 10 kGy (measured with a Fricke dosimeter). The experiment involved approximately 10 hours of exposure time to high-energy radiation. We used glass sealed ampoules under vacuum, containing approximately 250 mg of P(3HB) and 3 mL of VAc in bulk. The product was washed with acetone to eliminate the ungrafted PVAc and afterwards it was dried to constant weight at 50 °C in a vacuum oven. Subsequently, the graft copolymer P((3HB)-g-VAc) was hydrolysed in methanol solutions of 0.05 M of sodium hydroxide (NaOH) for approximately 10 hours to yield P((3HB)-g-VA). In the latter case, the grafted P(3HB) was also dried to constant weight. The powder obtained was grounded to a mesh size of 200 (74 μm). Sodium acetate (NaAc) (J.T. Baker) (74 μm) was used as pore former, and 0.3828 g of the graft copolymer was mixed with 0.1148 g of hydroxylated resin (Reichhold Química, Mex.), 0.0287 g of polyisocyanate and 0.2297 g of the porogen. The homogeneous dough was loaded into a stainless steel mould that allowed applying a pressure of approximately 5 MPa for 15 minutes. We obtained round shape scaffolds of roughly 1 cm in diameter and 2.5 mm in thickness. The obtained scaffolds were aerated for 24 hours and then filed down in order to expose the pores. The salt was leached from the scaffold by Sohxlet extraction in water for 24 hours. The morphology of the porous foam, previously coated with gold, was surveyed with SEM (JEOL-JSM-6060LV) operated at 15 kV. The compressive properties of the obtained scaffolds were estimated using an Instron mechanical testing machine; model Adamel Lhomargy DY.22 with a load cell of about 1 KN at a crosshead speed of 0.5 mm/min. The maximum load helped to determine the compressive strength while the compressive modulus was obtained from the slope of the initial linear region in the stress/strain curve. Ten specimens were tested to ensure reproducibility. All data presented herein was reported as mean ± standard and the statistical analysis was carried out using one-way analysis of variance (ANOVA). P values < 0.05 were considered as statistical significant (n = 15 for cell attachment and proliferation; n = 10 for mechanical and porosity test). Ten scaffolds were chosen for porosity testing. We used a method previously reported that involved filling a density bottle with ethanol. Then, the bottle was weighted (*m*_1_) in an analytical balance (Sartorius; Sartorius AG, Göttingen, Germany). Afterwards, the bottle is weighted while containing the scaffold and filled with ethanol (*m*_2_), and also the dry scaffold (*m*_*s*_ ) was weighted. Finally, the porosity (*φ*) is measured by the following equation 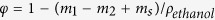, where 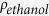 is the density of ethanol^42^.

### Primary cerebellar cultured astrocytes

All experimental procedures were conducted in accordance to the ethical polices for animal care and handling of the National University of Mexico. All experimental protocol was approved by the Instituto de Neurobiología Animal Care and Use Committee. Two male p5 CD1 mice were decapitated for each; their brains were rapidly removed and placed in a Petri dish with cold phosphate buffer solution (PBS) (137 mM NaCl, 2.7 mM KCl, 10 mM Na_2_HPO_4_ • 2 H_2_O, 2 mM KH_2_PO_4_; pH 7.4). The cerebellum was isolated in a cold PBS buffer, chopped into small pieces and placed in an Eppendorf tube containing 200 μl of Dulbecco’s Modified Eagles Medium (DMEM) supplemented with 10% fetal bovine serum; 2 mM glutamine, 100 UI/ml penicillin and 100 μg/ml streptomycin. The small pieces of cerebellum were mechanically dissociated into individual cells by several passes through a fire polished Pasteur pipette tip, previously treated with Sigmacote^®^ (SIGMA*). The supernatant was suspended into an Eppendorf tube with 500 μl of DMEM. The cell suspension was plated on sterilised P1M3DH scaffolds and diluted with 3 ml of DMEM in a 35 mm Petri dish. The cultures were kept five days *in vitro* (5 DIV) at 37 °C under a mixed air and 5% CO_2_ atmosphere. The medium was changed every 2 days^52^,^53^. Twenty-four hours before confocal imaging, the cells were transduced with mCherry using an adenoviral vector (All culture reagents were purchased from Gibco BRL).

### Loading cells for calcium imaging

P1M3DH scaffolds were sterilised under UV light for 10 min and glued on the surface of a Petri dish, then HEK293 cells were placed on top of the scaffolds and maintained in Dulbecoo’s Modified Eagle Medium M (Gibbco^TM^) containing 10% fetal bovine serum and antibiotics (100 UI/ml penicillin and 100 UI/ml streptomycin). After two days at 37 °C and 5% of CO_2_, cells were loaded for 30 min, with the calcium indicator Fluo-4AM (Molecular Probes^®^) dissolved in 50 μl DMSO containing 20% Pluronic F-1227 (SIGMA*) and further diluted in a calcium free dye buffer (125 mM NaCl, 2 mM MgCl_2_, 4.5 mM KCl, 10 mM Glucose, 20 mM HEPES) to yield a final concentration of 0.5 mM Fluo-4AM. Dynamics of cytosolic Ca^2+^ were monitored by imaging changes of fluorescence intensity before and after adding to the medium the high potassium solution (140 mM KCl, 2 mM MgCl_2_, 20 mM, 2 mM CaCl_2_, Glucose, 10 mM HEPES). Time lapse confocal recordings (Zeiss LSM510) were taken for 1.5 s at 1.5 Hz using a wavelength of 488 nm for excitation, reconstructed with *ImageJ* and analysed with MATLAB.

### Data analysis

Frames of 512 × 512 pixels were acquired at 1.5 Hz. Image sequences were analysed with custom programmes written in MATLAB. Cell-outlines were detected using a semi-automated algorithm based on cell fluorescence intensity, cell size and shape. After labeling the cell-based regions of interest (ROIs) all pixels within each ROI were averaged to give a single time course (∆F/F).

## Additional Information

**How to cite this article**: Reyes, A. P. *et al*. Novel Poly(3-hydroxybutyrate-g-vinyl alcohol) Polyurethane Scaffold for Tissue Engineering. *Sci. Rep.* **6**, 31140; doi: 10.1038/srep31140 (2016).

## Supplementary Material

Supplementary Information

Supplementary Video
