# Supplementary material for: Novel Poly(3-hydroxybutyrate-g-vinyl alcohol) Polyurethane Scaffold for Tissue Engineering
Source: Sci Rep. 2016 Aug 9;6:31140. doi: 10.1038/srep31140 (PMC4977462; doi:10.1038/srep31140)
Supplement: Supplementary Information [file srep31140-s1.pdf]

Novel Poly(3-hydroxybutyrate-g-vinyl alcohol) Polyurethane Scaffold for Tissue Engineering

Adriana Pétriz Reyes [1], *Ataúlfo Martínez-Torres* [1], *Ma. del Pilar Carreón-Castro* [2], *José Rogelio Rodríguez Talavera* [3], *Susana Vargas Muñoz* [3], *Víctor Velázquez Aguilar*[4], *Maykel González Torres* [2]*

[1] *Laboratorio de Neurobiología Molecular y Celular, Instituto de Neurobiología, Universidad Nacional Autónoma de México, Querétaro 76230, México.*
[2]*Instituto de Ciencias Nucleares, Universidad Nacional Autónoma de México, México D.F, 04510., México.*
[3]*Centro de Física Aplicada y Tecnología Avanzada, Universidad Nacional Autónoma de México, Querétaro 76230, México.*
[4]*Facultad de Ciencias, Universidad Nacional Autónoma de México, México D.F, 04510., México.*

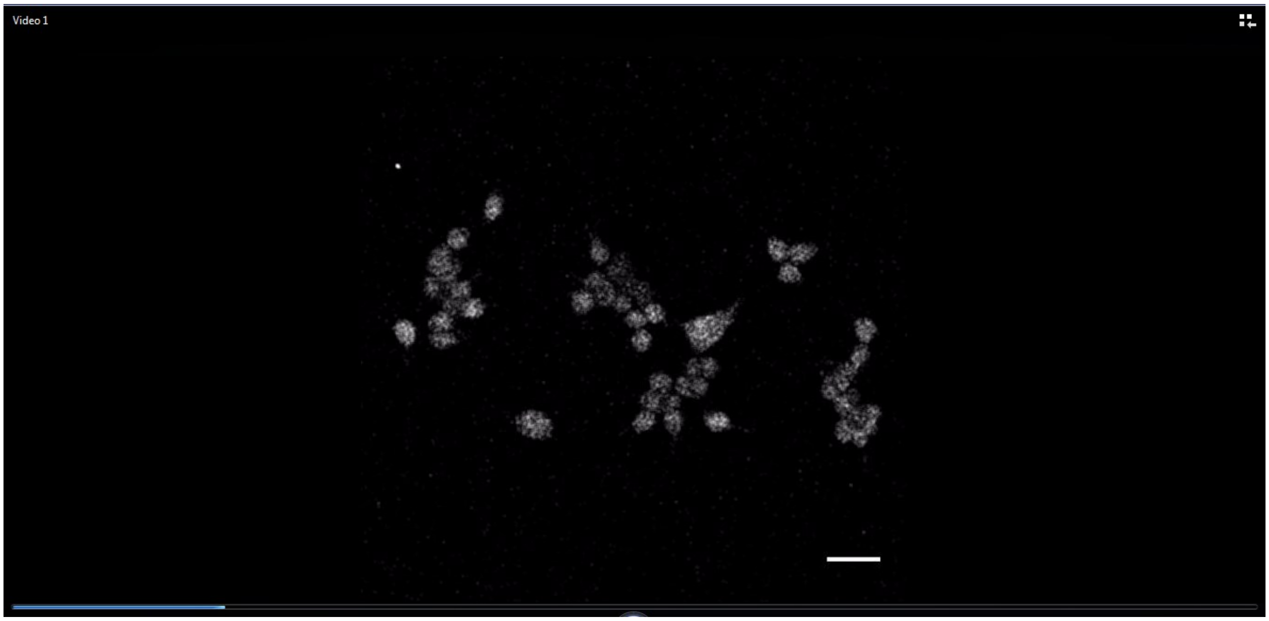

Video 1. Dynamics of cytosolic Ca^{2+} monitored by imaging changes of fluorescence intensity before and after adding to the medium the high potassium solution (Time lapse confocal recordings of HEK293 cells).
